# Delivery of liothyronine by microneedle patch for the treatment of hypothyroidism

**DOI:** 10.1007/s13346-026-02214-6

**Published:** 2026-09-02

**Authors:** Yasmine Gomaa, Tasnuba Hasin, Martin N. Griffin, Thy C. Nguyen, Richard K. Noel, Laura O’Farrell, Elizabeth A. McAninch, Ann Marie Kaiser, Levi B. Wood, Mark R. Prausnitz

**Affiliations:** 1 School of Chemical and Biomolecular Engineering, Georgia Institute of Technology, Atlanta, GA USA; 2 Department of Pharmaceutics, Faculty of Pharmacy, Alexandria University, El-Khartoum Square, Alexandria, Egypt; 3 George W. Woodruff School of Mechanical Engineering, Georgia Institute of Technology, Atlanta, GA USA; 4 School of Chemistry and Biochemistry, Georgia Institute of Technology, Atlanta, GA USA; 5 Department of Animal Resources, Georgia Institute of Technology, Atlanta, GA USA; 6 Division of Endocrinology, Gerontology, and Metabolism, Department of Medicine, Stanford University School of Medicine, Stanford, CA USA; 7 Equilibrate Therapeutics, LLC, Santa Barbara, CA USA

**Keywords:** Liothyronine, Microneedle patch, Pharmacokinetics, Hypothyroidism, Thyroid hormone, Intradermal drug delivery

## Abstract

Hypothyroidism is conventionally treated with thyroid hormone replacement *via* synthetic hormones, namely levothyroxine (LT_4_), and in possible combination with liothyronine (LT_3_), both administered orally. The supratherapeutic peaks and subtherapeutic troughs of LT_3_ concentration after oral delivery may limit efficacy, as this pharmacokinetic profile does not mimic the relatively constant T_3_ levels found in normal physiology. Microneedle patches (MNPs) provide a simple-to-use delivery method that can be formulated to achieve controlled drug release for extended periods of time. Here, we developed a MNP for LT_3_ delivery into the skin as a first assessment of this approach. After examining a number of MNP formulations, we developed MNPs containing a range of different LT_3_ doses in a poly(vinylpyrrolidone)-based formulation. The MNs effectively punctured the skin and achieved average LT_3_ delivery efficiencies of 41–71%. LT_3_ MNP administration to hypothyroid rats at three doses (10 µg, 100 µg, 1000 µg) produced elevated serum T_3_ levels that persisted for 18–24 h, exhibiting a nonlinear dependence on LT_3_ dose. This study established the feasibility of administering LT_3_ by MNP that could be translated to enable improved therapy for patients with hypothyroidism.

**Graphical Abstract:**

**Figure:**
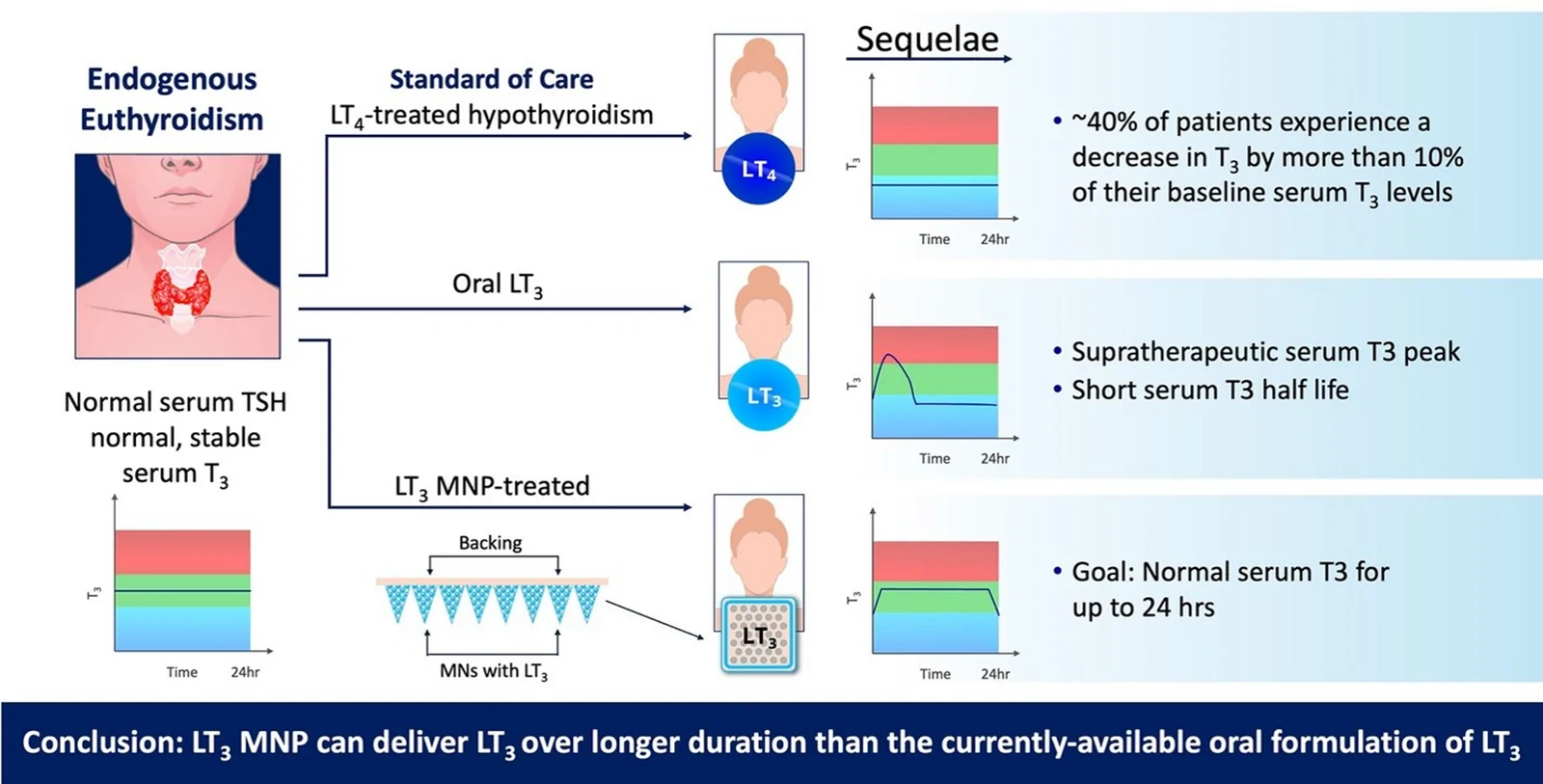

**Supplementary Information:**

The online version contains supplementary material available at https://doi.org/10.1007/s13346-026-02214-6.

## Introduction

Thyroid hormone homeostasis plays essential roles in growth, development, cognition, mood, energy expenditure, and thermoregulation [1]. A healthy thyroid gland secretes both thyroxine (T_4_) and triiodothyronine (T_3_), where T_4_ is considered the prohormone and T_3_ the active form, as it exhibits 10–30 times greater affinity for the thyroid hormone receptor [2].

A condition called hypothyroidism occurs when the thyroid gland cannot produce normal amounts of thyroid hormone. Hypothyroidism is common, affecting over 11% of the United States population [3, 4]. The standard of care for treatment of hypothyroidism has been synthetic levothyroxine (LT_4_) administered at doses sufficient to normalize serum thyroid-stimulating hormone (TSH) [1, 5]. When LT_4_ is administered orally, it has a long half-life and maintains stable serum levels of T_4_ [1]. Because of the high prevalence of hypothyroidism and its ability to normalize serum TSH levels, LT_4_ is consistently one of the most prescribed drugs in the world [6].

LT_4_-treated patients with normal serum TSH, however, may exhibit (i) relatively lower serum T_3_ levels, (ii) relative hypometabolism compared to healthy controls, and (iii) persistent symptomatology including fatigue, difficulty maintaining body weight, depressed mood, and cold intolerance [5, 7, 8]. Specifically, at least 20% of LT_4_-treated patients with hypothyroidism still suffer from impairment in psychological well-being compared to healthy controls [9]. To address this issue some, but not all, clinical trials have shown improved outcomes with LT_4_ + liothyronine (LT_3_) “combination therapy” [10]. Notably, these studies have all been performed with the currently FDA-approved formulation of oral LT_3_, but its pharmacokinetic (PK) profile does not mimic physiologic, endogenous T_3_ secretion by the intact thyroid gland [10, 11]. Whereas T_3_ secreted by the thyroid is maintained in remarkably stable serum levels [12], LT_3_ administered orally has several drawbacks including supratherapeutic serum peak about 2–4 h after ingestion [13] and short half-life [11, 14] To best maintain normal serum T_3_ levels, LT_3_ needs to be taken multiple times daily [11]. Despite the imperfections associated with oral LT_3_, thyroid professional societies now endorse the option of LT_4_ + LT_3_ combination therapy for patients with hypothyroidism and residual symptoms during LT_4_ monotherapy [1, 10, 15, 16].

Multiple investigators have attempted to develop an improved LT_3_ formulation with a PK profile that better mimics the stable T_3_ secretion in euthyroidism [17]. For example, orally administered metal-coordinated Poly-Zinc-Liothyronine (PZL) in rodents [18] and humans [19], and orally administered triiodothyronine sulfate [20] have both improved PK over currently available LT_3_ preparations. Compounded topical LT_3_ does not effectively penetrate the skin’s *stratum corneum* barrier and, even if it did, the skin contains the deactivating type 3 deiodinase (D3) that converts T_3_ to an inactive metabolite, T_2_ [21–23].

**Fig. 1:**
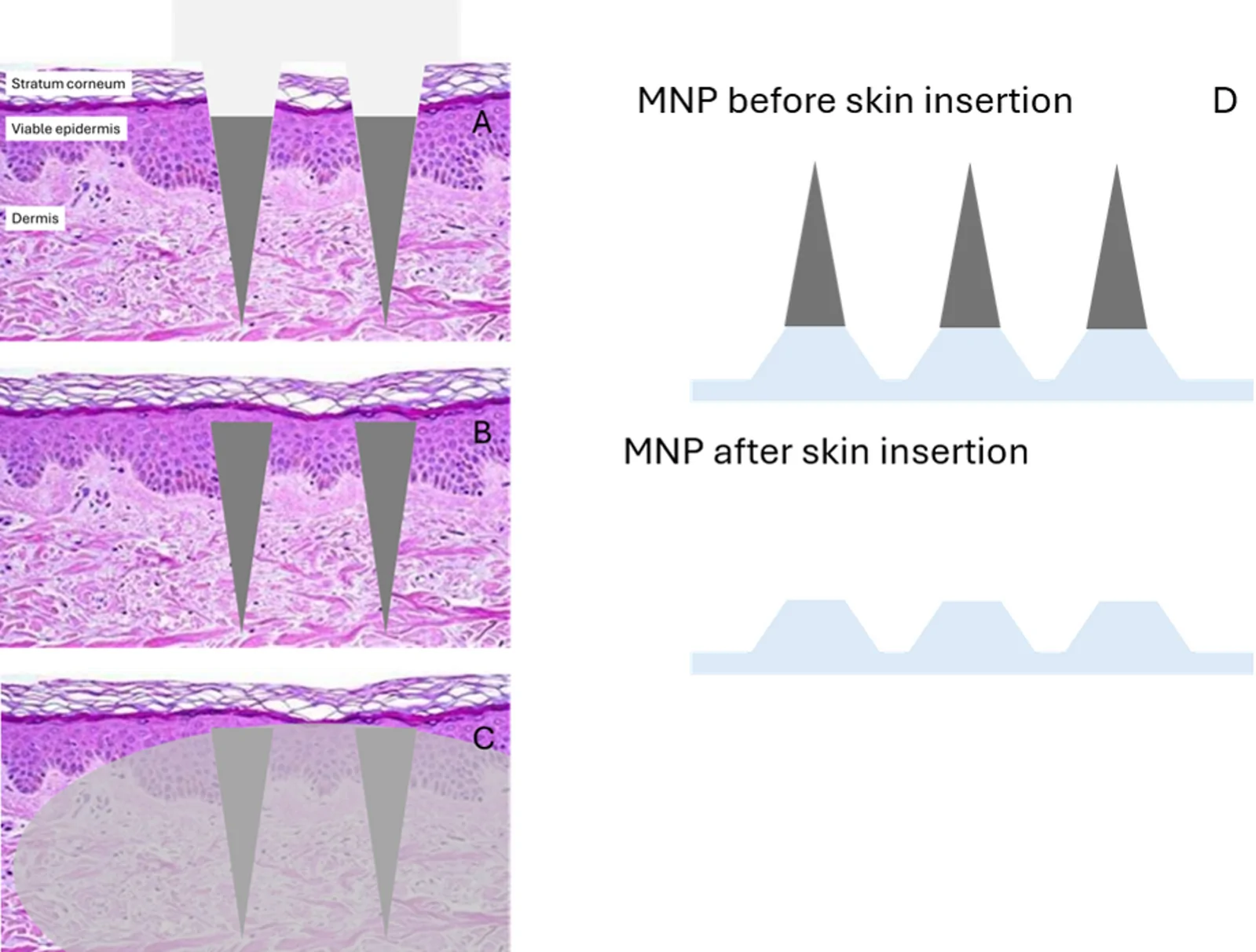
Schematic drawing of LT_3_ delivery by MNP. (**A**) MNP is applied to the skin. (**B**) MNs dissolve off patch backing and remain embedded in the skin. (**C**) LT_3_ is released from MNs in the skin for capillary uptake and systemic distribution. (**D**) MNP before and after insertion in skin showing MN dissolution

To overcome the low bioavailability of topically applied LT_3_ and facilitate sustained delivery over time, we investigated the formulation and delivery of LT_3_ by microneedle patch (MNP) as illustrated in Fig. 1. Microneedles (MNs) are solid projections that painlessly penetrate across the *stratum corneum* barrier of the skin to deposit drug in the deeper epidermis and dermis [24]. MNs can be formulated to encapsulate drug for rapid dissolution to achieve bolus delivery or with formulations that enable slow drug release for up to months in duration [25, 26]. MNPs can be easily applied to the skin with little or no training, thereby facilitating patient access through self-administration [27]. MNPs are in widespread use as consumer products for cosmetic applications [28], and have been studied in dozens of clinical trials for the delivery of drugs, vaccines and other actives [29, 30].

In this study, we sought to develop MNPs for the delivery of LT_3_. To assess their suitability for LT_3_ administration as a future treatment of hypothyroidism, we formulated MNPs to contain targeted amounts of LT_3_, studied their delivery of LT_3_ into skin ex vivo and in vivo, and assessed LT_3_ PK in a hypothyroid rat model.

## Materials and methods

### Materials

LT_3_ was obtained from Hubei Vanzpharm (Wuhan City, China). Analytical grade polyvinyl alcohol (PVA 4–88) was purchased from Polysciences (Warrington, PA). LT_3_ has a molecular weight of 651 Da and moderately lipophilic character (XLogP3-AA = 1.7) [31]. All other excipients with analytical grades were obtained from Sigma-Aldrich (St. Louis, MO). Gentian violet solution (2% w/v) was purchased from Humco (Texarkana, TX).

### Manufacturing LT_3_ microneedle patches

MNPs were manufactured by a two-step micromolding process involving a first cast of LT_3_ solubilized or suspended in a polymeric solution to form the MNs (10 × 10 array of 600 μm long microneedles, 150 μm in base radius, 1000 μm inter-needle spacing, a sharp tip of ~ 10 μm in radius and an area of 1 cm^2^) followed by a second cast of another polymeric solution to form the backing of the MNP of ~ 1 mm in thickness. Several formulations were used for the first-cast solution that (i) achieved different LT_3_ loads per MNP, (ii) employed different polymers – i.e., polyvinyl alcohol (PVA) and sucrose or polyvinylpyrrolidone (PVP) of different molecular weights, and (iii) were cast using either deionized (DI) water or absolute ethanol as solvent (Table 1). The second-cast solution was composed of 18% w/v of PVA and 18% w/v sucrose in DI water.

**Table 1:** Formulations of first-cast solution used to make LT_3_ MNPs

Code	Expected LT_3_ load per MNP (µg)	First-cast solution composition (w/v)	Solvent
Study 1 (in vitro)			
MNP A	400	3% PVP 360 kDa	Ethanol
MNP B	700	3% PVP 360 kDa	Ethanol
MNP C	400	10% PVA / 10% sucrose	Water
MNP D	700	10% PVA / 10% sucrose	Water
Study 2 (in vitro)			
MNP E	667	10% PVP 10 kDa	Water
MNP F	667	10% PVP 55 kDa	Water
MNP G	667	10% PVP 360 kDa	Water
Study 3 (in vitro)			
MNP H	667	5% PVP 10 kDa	Water
MNP I	667	5% PVP 55 kDa	Water
MNP J	667	5% PVP 360 kDa	Water
Study 4 (in vitro)			
MNP K	300	5% PVP 10 kDa	Water
MNP L	600	5% PVP 10 kDa	Water
Study 5 (in vivo)			
MNP M	10	5% PVP 10 kDa	Water
MNP N	100	5% PVP 10 kDa	Water
MNP O	1000	5% PVP 10 kDa	Water

To make LT_3_ MNPs, 10 µl of a first-cast solution was filled in the conical cavities of a polydimethylsiloxane (PDMS) mold containing MN cavities arranged in a 10 × 10 array, which was then centrifuged at 3234 g for 1 h at 25 °C (Eppendorf 5804 R centrifuge, swinging bucket rotor S-4-72, Hamburg, Germany) to fill the mold cavities and partially dry the first-cast solution. The molds were then filled with 250 µl of the second-cast solution with vacuum maintained for 2 h at room temperature (20–25 °C), followed by drying for 3 days at 25 °C with desiccant. Manufactured MN patches were then stored at 25 °C with desiccant until further use.

### Quantification of LT_3_ content by HPLC

LT_3_ content in MNPs was determined by dissolving MNPs in a mixture (50:50 v/v) of 0.02 M sodium hydroxide in DI water and methanol, and measuring LT_3_ concentration in the resulting solution by reverse-phase high-performance liquid chromatography (HPLC) using an Agilent 1200 series instrument and an Eclipse Plus C18 column (4.6 × 250 mm, 5 μm) from Agilent Technologies (Santa Clara, CA). Although we did not determine lower limits of detection and quantification, we found that the calibration curve was linear down to 2 µg/mL LT_3_ (Fig. S1A in Supplementary Information) and therefore used that as our effective lower limit of quantification. Mobile phases were prepared by mixing 0.1% trifluoroacetic acid in HPLC-grade water with acetonitrile at a volumetric ratio of 30:70. The flow rate was set to 1 mL/min, and the column temperature was set to 25 °C. The injection volume was 10 µl and the detection wavelength was 225 nm. LT_3_ concentration was determined from a generated calibration curve of known concentrations of LT_3_ in 0.02 M methanolic sodium hydroxide (50:50 v/v) solution (Fig. S1A in Supplementary Information).

### Insertion of LT_3_ MNPs into skin ex vivo

To assess their mechanical strength, LT_3_ MNPs were inserted in pig skin ex vivo (obtained from slaughterhouse) by thumb equivalent to a force of approximately 20 N. After 15 min, the patches were removed. Gentian violet solution was then applied to the treated skin site for 5 min, after which the remaining dye was cleaned from the skin surface using alcohol wipes, and the skin was examined microscopically (Hirox KH-8700, Tokyo, Japan) for dye staining of pores created by MN puncture into the skin. Microneedle insertion efficiency was determined as the percentage of needles that successfully punctured the skin (indicated by gentian violet-stained pores) relative to the total number of microneedles per patch. The used MNPs were also inspected microscopically to image the extent of MN dissolution.

### LT_3_ delivery from MNPs into skin ex vivo

Pig skin (0.8–1.4 mm thick) was mounted on stainless steel mesh (Valchoose, China) cut into a circle of 33 mm diameter and both were glued to the rim of the receiver chamber of a Franz diffusion cell (PermeGear, Bethlehem, PA). The receiver chamber was filled with 10 mL of phosphate-buffered saline (PBS) with 50 mM sodium azide and placed in a stirred heating block (HS-2; PermeGear) at 37 ± 0.5 °C with continuous magnetic stirring (400 rpm). After allowing the skin to equilibrate in the Franz cell for 1 h, LT_3_ MNPs were pressed to the skin by thumb application and kept in place for up to 1 week by a 50-gram weight. Aliquots of 100 µL were collected from the receiver chamber at predetermined times for up to 1 week and replaced with the same volume of fresh PBS. Each aliquot was syringe-filtered with a 0.22 μm filter (VWR) and LT_3_ content was determined by HPLC.

### LT_3_ pharmacokinetics in hypothyroid rats

In addition to assessing LT_3_ delivery in cadaver skin, we sought to establish whether LT_3_ could be delivered from a MNP into the systemic circulation in vivo. Male Wistar rats (10 weeks old, ~ 320 g, Charles River Laboratories, Wilmington, MA) were acclimatized for at least 6 weeks during which animal weight, water and food intake were monitored. After acclimatization, hypothyroidism was induced by adding methimazole (0.05% w/v) to drinking water for 4 weeks [32]. Hypothyroidism was maintained by feeding animals a low-iodine diet (TD.120460, Inotiv, Lafayette, IN) for 6 weeks. Other investigators have previously published results utilizing oral LT_3_ control rodents under a similar model so an oral control group was deferred in these LT_3_ MNP proof-of-concept studies [18]. Two days before MNP application to the rats, a chronic jugular catheter was aseptically implanted under isoflurane inhalation anesthesia (Covetrus, Dublin, OH) to facilitate repeated sampling. Catheters were flushed once daily with 100 units/mL of heparinzed saline (Sagent Pharmaceuticals, Schaumburg, IL) prior to MNP application and after each sampling. See Note 1 in Supplementary Information for details.

Before MNP application, a section of hair was clipped from the posterior side of the neck of the rat with an area of approximately 10 cm^2^ under isoflurane anesthesia, followed by a brief application of depilatory cream (Nair, Princeton, NJ) that was cleaned off with gauze and DI water. MNPs containing doses of 10, 100, or 1000 ug LT_3_ were then pressed by thumb to the hairless region of skin. Patches were held in place throughout the experiment (48 h) using a Tegaderm film (3 M Health Care, Saint Paul, MN) secured with a harness (Rat VAH harness, Instech, Plymouth Meeting, PA). LT_3_ Doses in the MN patches were selected to cover a broad range to elucidate dose-response relationship taking into consideration not to exceed the LT3 LD50 (20 mg/Kg) in rats [33].

Blood (600 µl) was collected and incubated overnight at 4 °C, after which serum was separated by centrifugation (Eppendorf 5415 R centrifuge, fixed bucket rotor F45-24-11) at 1000 g for 15 min at 4 °C and stored at -80 °C until analysis. LT_3_ content in collected serum was determined using an enzyme-linked immunosorbent assay (Rat Tri-iodothyronine (T3) ELISA Kit, CUSABIO, Houston, TX) or a Luminex multiplexed immunoassay (MILLIPLEX Rat Thyroid Magnetic Bead Panel - Endocrine Multiplex Assay, MilliporeSigma, Burlington, MA) using methods described in Note 2 in Supplementary Information. At the end of the study, animals were euthanized by carbon dioxide asphyxiation and skin at the sites of MNP application was excised and subjected to LT_3_ extraction for LT_3_ content determination using HPLC as described above. All animal studies were approved by the Georgia Tech Institutional Animal Care and Use Committee (IACUC).

### LT_3_ delivery efficiency into skin

To assess delivery efficiency of LT_3_ administered by MNPs into skin, MNPs were pressed by thumb into pig skin (ex vivo) or rat skin (in vivo), and left on the skin for a predetermined time. A single tape stripping with a Tegaderm Film was used to collect LT_3_ deposited on the skin surface during MNP application, followed by LT_3_ extraction and quantification using HPLC, as described above. The computed amount of LT_3_ delivered to skin was calculated by subtracting the residual amount of LT_3_ present in MNPs after use and the LT_3_ collected from the skin surface after skin insertion from the initial LT_3_ load in the MNPs, as determined by HPLC. The actual amount of LT_3_ delivered to skin was determined by extracting LT_3_ from the skin at the MNP application site using 0.02 mM NaOH in methanol followed by HPLC analysis.

### Statistical analysis

Statistical analysis was performed using GraphPad Prism software version 10.0.0 for Windows (GraphPad Software, Boston, MA). Comparisons between individual samples were done either using a Mann-Whitney U Test or ANOVA. Probability (p) values of < 0.05 were considered to be significant.

## Results

This study sought to assess the feasibility of using MNPs to administer LT_3_ into the systemic circulation *via* the skin for potential future use in the treatment of hypothyroidism. Because LT_3_ MNPs have not been studied before, our first goal was to study formulations that effectively incorporate LT_3_. We had an additional goal of using a water-soluble formulation that would rapidly dissolve (e.g., in minutes) into the skin, thereby minimizing the patch wear time on the skin before MN dissolution, and would interact with LT_3_ (of a slower dissolution) to help form a drug depot in the skin to enable the slow LT_3_ release for hours. 

### **MNP formulation optimization and in vitro skin permeation**

In study #1, we initially screened two types of casting solutions, specifically an organic (MNP A and MNP B; Table 1) versus an aqueous (MNP C and MNP D; Table 1) casting solution, each containing different polymers (PVP or PVA) at two different LT_3_ loadings (400 or700 µg per MNP). In formulations MNP A and MNP B, PVP was utilized because PVP: (i) is soluble in organic solvents into which LT_3_ can be solubilized, specifically absolute ethanol [34], (ii) has been shown to provide sufficient mechanical strength to serve as a MN matrix material [35, 36], and (iii) is widely used in pharmaceutical formulations [37]. We therefore made MNs using a casting solution containing PVP with a high molecular weight (360 kDa) to promote mechanical strength at a 3% (w/v) concentration in ethanol. In formulations MNP C and MNP D, we used a LT3 suspension in an aqueous polymeric solution of PVA and sucrose due to its common use to make strong MNs [38–40]. In both formulations, LT_3_ was incorporated into the MNPs at two different LT3 loadings (400 or700 µg per MNP).

**Fig. 2:**
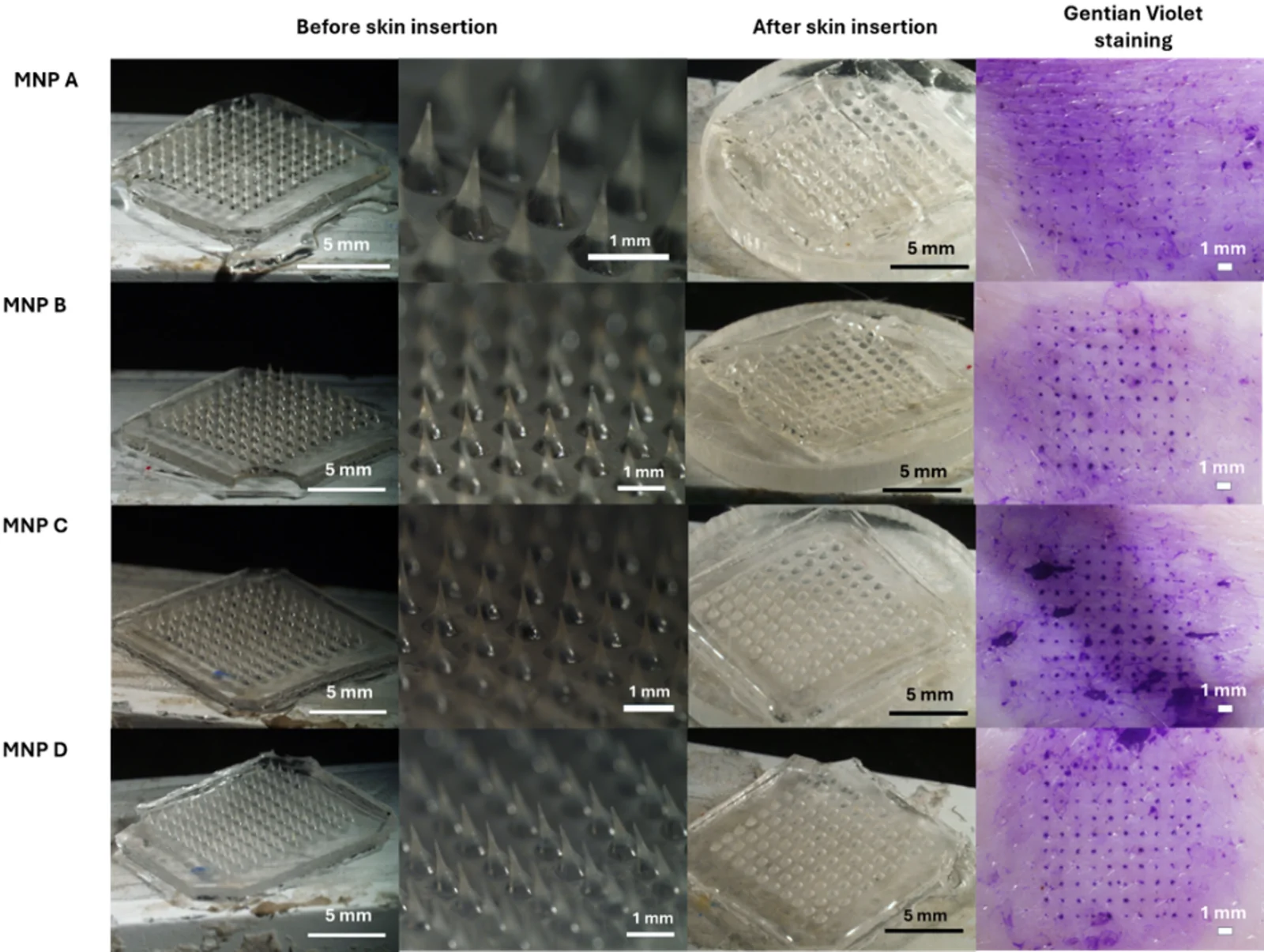
MNPs containing LT_3_ and insertion testing into pig skin ex vivo. Representative MNPs are shown at two magnification levels imaged by light microscopy before and after insertion into skin with 15 min skin wear time using MNP formulations MNP A, MNP B, MNP C and MNP D (see Table 1). Representative images are also shown of skin stained with gentian violet to identify pores created at MN puncture sites

**Fig. 3:**
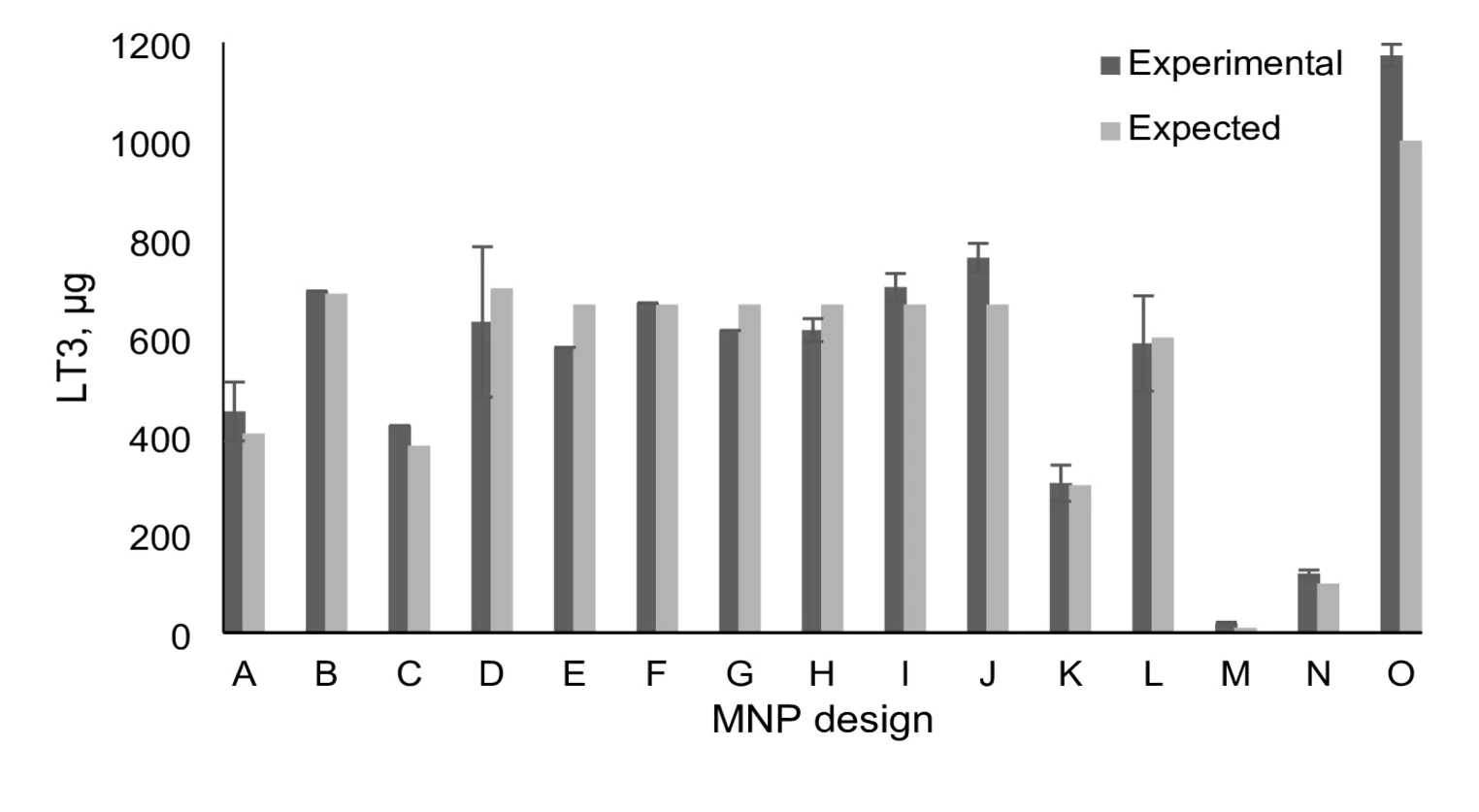
LT_3_ content of MNPs. Experimental LT_3_ values were determined by HPLC measurement of MNP contents after reconstitution by dissolution. Expected LT_3_ values were determined by calculating the amount of LT_3_ in 10 µl of casting solution. MNP formulations were MNP A through MNP O (see Table 1). Data represent mean ± SD of *n* = 3 replicates

All tested formulations produced intact microneedles, with 100% demolding success rate as characterized microscopically (Fig. 2) with the expected LT_3_ loading (Fig. 3). No impact of MNP storage or handling on MN integrity was noted. However, we noticed LT_3_ precipitation upon drying into the MNs of formulations MNP A and MNP B (Table 1) by visual observation, despite LT_3_ having been solubilized in the casting solution initially (Fig. 2). Ex vivo pig skin assessment of MN insertion and MN dissolution testing showed successful insertion for all MNP formulations as indicated by gentian violet staining of the MN created pores with 100% insertion efficiency (Fig. 2). Partial MN dissolution was seen after 15 min skin wear time.

Given the successful fabrication of MNPs with an aqueous casting solution, we decided to avoid using organic casting solutions due to possible LT_3_ precipitation seen in MNP A and MNP B, and the need to remove residual solvent from the final product. Because of the hypothesized interaction between PVP and LT_3_ to enable depot formation in the skin for extended drug release, we modified the aqueous casting solution formulation to use PVP as the MN matrix material. The incomplete dissolution of MNs seen after skin insertion (Fig. 2) suggested the need to consider PVP of lower molecular weight to facilitate MN dissolution, but still achieve sufficient mechanical strength for skin insertion.

Based on these considerations, in study #2, aqueous casting solutions were formulated containing PVP at a concentration of 10% (w/v) using three different PVP molecular weights (10 kDa for MNP E, 55 kDa for MNP F, and 360 kDa for MNP G; Table 1). All three of these MNP formulations produced well-formed MNs (Fig. S2 in Supplementary Information) in agreement with expected LT_3_ loading (Fig. 3). However, upon testing LT_3_ release from these MNPs in pig skin ex vivo using Franz diffusion cells, we could not detect LT_3_ in the receiver chamber of any of the tested MNPs after 24 h. We hypothesized that a lower concentration of PVP (5% versus 10% (w/v)) and a longer delivery period (48 h versus 24 h) might be needed to accommodate the dissolution and diffusion of LT_3_ through the skin in the Franz cell.

**Fig. 4:**
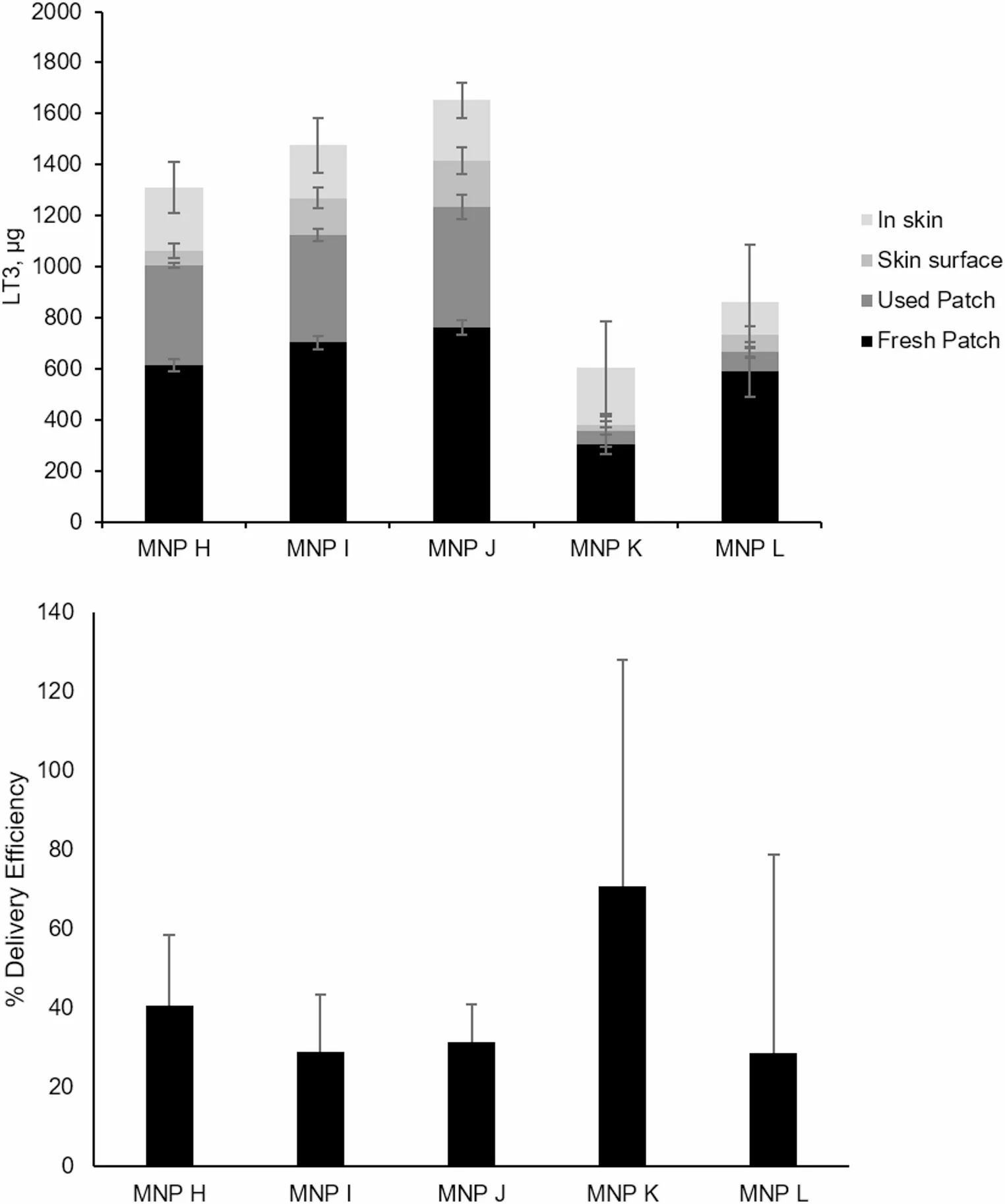
LT_3_ delivery efficiency from MNPs into pig skin ex vivo. MNPs with different formulations were applied to skin for 48 h for MNP H-J and 1 week for MNP K-L (see Table 1). (**a**) LT_3_ content in fresh MNPs and used MNPs was determined by MNP dissolution and LT_3_ measurement by HPLC. LT_3_ content on the skin surface was determined by tape-strip removal of LT_3_ from the skin surface, followed by LT_3_ extraction from the tape and analysis by HPLC. LT_3_ in skin was determined by extracting LT_3_ from the skin at the MN patch application site. (**b**) LT_3_ delivery efficiency was determined as the LT_3_ content in the skin divided by the LT_3_ content in a fresh patch. MNP formulations were MNP H through MNP L (see Table 1). Data represent mean ± SD of *n* = 3 replicates

In study #3, we formulated casting solutions with PVP at a concentration of 5% (w/v) at the same three PVP molecular weights (10 kDa for MNP H, 55 kDa for MNP I, and 360 kDa for MNP J; Table 1). The resulting MNPs were again all well-formed (Fig. S2 in Supplementary Information) and agreed with expected loading (Fig. 3). However, using the reduced PVP concentration and extending the duration of the in vitro permeation testing still did not result in any measurable permeation of LT_3_. When comparing skin delivery efficiency among the three MNPs, there was no significant difference (*p* > 0.05, Student’s t-test, Fig. 4). Based on the equivalent performance of the MNPs, we selected the MN casting formulation containing 5% (w/v) PVP with a molecular weight of 10 kDa as our favored composition based on the hypothetical expectation that it would enable faster dissolution in the skin.

**Fig. 5:**
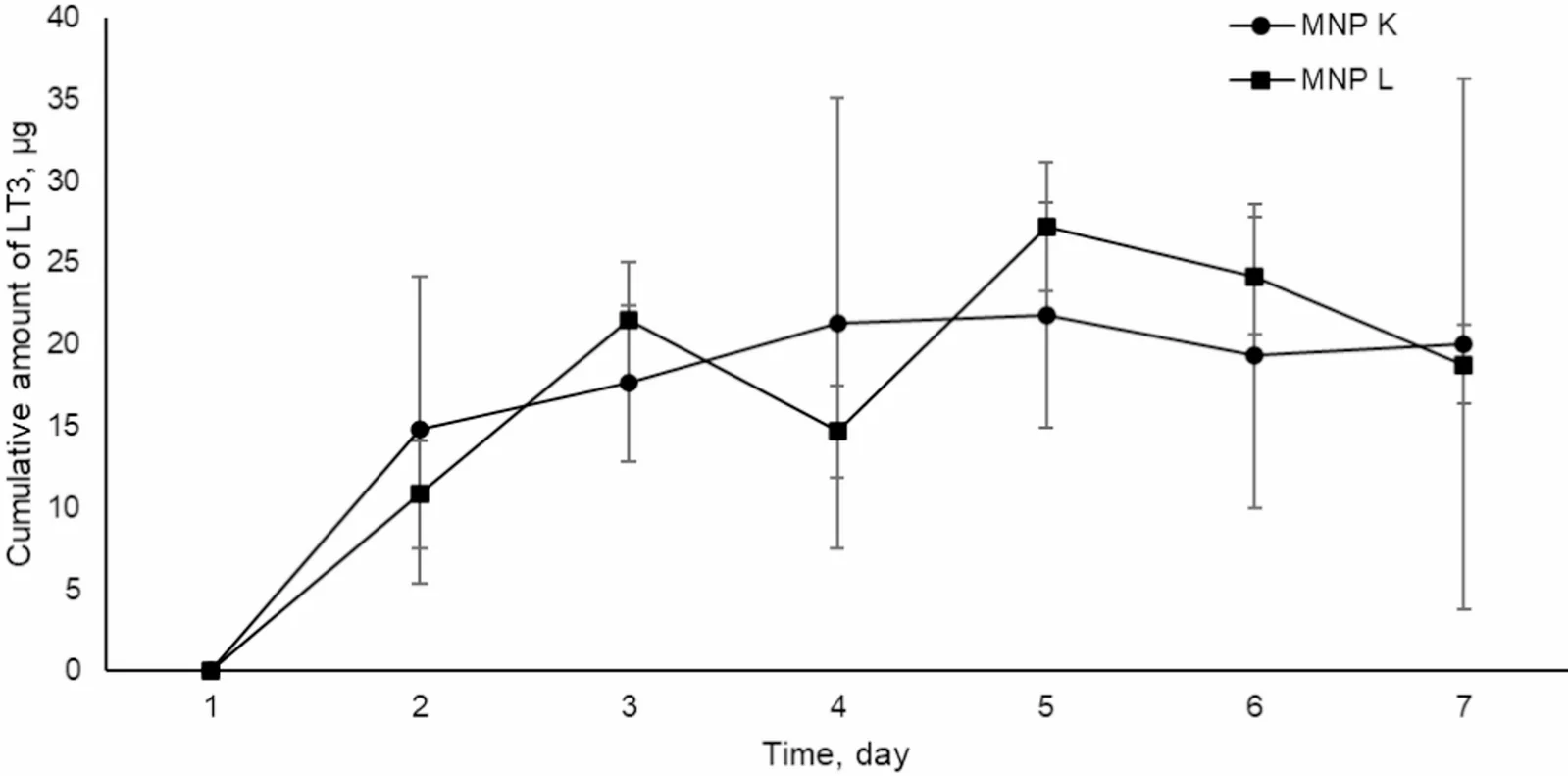
Transdermal Permeation of LT_3_ through pig skin ex vivo. The cumulative amount of LT_3_ permeated through skin after application of LT_3_ MNPs. MNP formulations were MNP K and MNP L (see Table 1). Data represent mean ± SD of *n* = 3 replicates

We next assessed the role of LT_3_ loading on drug release. In study #4, we made MNPs using casting solutions with different LT_3_ loadings (300 µg for MNP K, 600 µg for MNP L; Table 1) and further extended the in vitro permeation testing to one week. Both tested formulations again resulted in well-formed microneedles (Fig. S2 in Supplementary Information) with the expected loading (Fig. 3). In vitro skin permeation testing exhibited a lag time of one day for both formulations, presumably representing the time it took for LT_3_ to dissolve in the interstitial fluid of the dermis and diffuse through the dermis to reach the receiving solution in the Franz cell (Fig. 5, Fig. S4 in Supplementary Information). After that, there were measurable amounts of LT_3_ released through the skin during the 7-day experiment with no significant differences between the two formulations (*p* > 0.05, two-way ANOVA).

### Pharmacokinetics of LT_3_ in hypothyroid rats after MNP administration

**Fig. 6:**
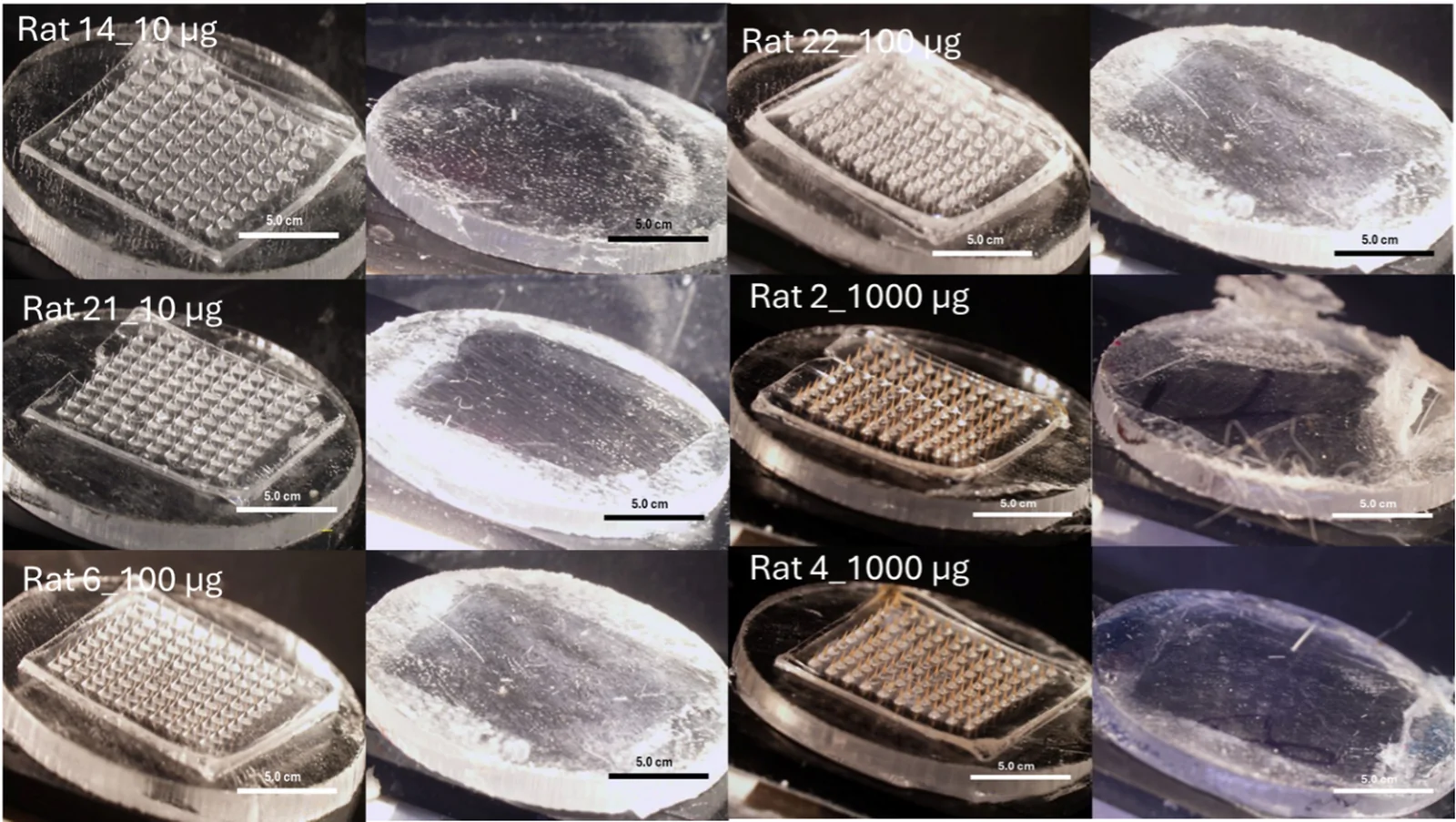
LT_3_ MNPs before and after insertion into rat skin in vivo. Representative MNPs are shown before and after insertion into skin for 48 h. Imaging was performed by light microscopy. MNP formulations were MNP M (10 µg LT_3_, rats 14 and 21), MNP N (100 µg LT_3_, rats 6 and 22), and MNP O (1000 µg LT_3_, rats 2 and 4) (see Table 1)

Guided by ex vivo findings, we next evaluated LT_3_ delivery in vivo using a hypothyroid rat model over a range of LT_3_ doses. We made MNPs using the 5% (w/v), 10 kDa PVP formulation loaded with target LT_3_ doses of 10 µg (MNP M), 100 µg (MNP N) and 1000 µg (MNP O) to use in a PK study (Table 1). Low molecular weight PVA (10 kDa) was used in part due to its safety and rapid clearance from the body through the kidneys [41]. These MNPs were well-formed after fabrication, generally contained LT_3_ in agreement with the expected loading, and exhibited complete microneedle dissolution upon application to rat skin in vivo indicating a penetration depth of approximately 600 μm (Fig. 6).

We quantified LT_3_ delivery into rat skin in vivo and determined that MNP N delivered 65 ± 20 µg LT_3_ and MNP O delivered 533 ± 334 µg LT_3_, corresponding to a delivery efficiency of 38% and 53%, respectively. The delivered amount of LT_3_ from MNP M was not measured.

**Fig. 7:**
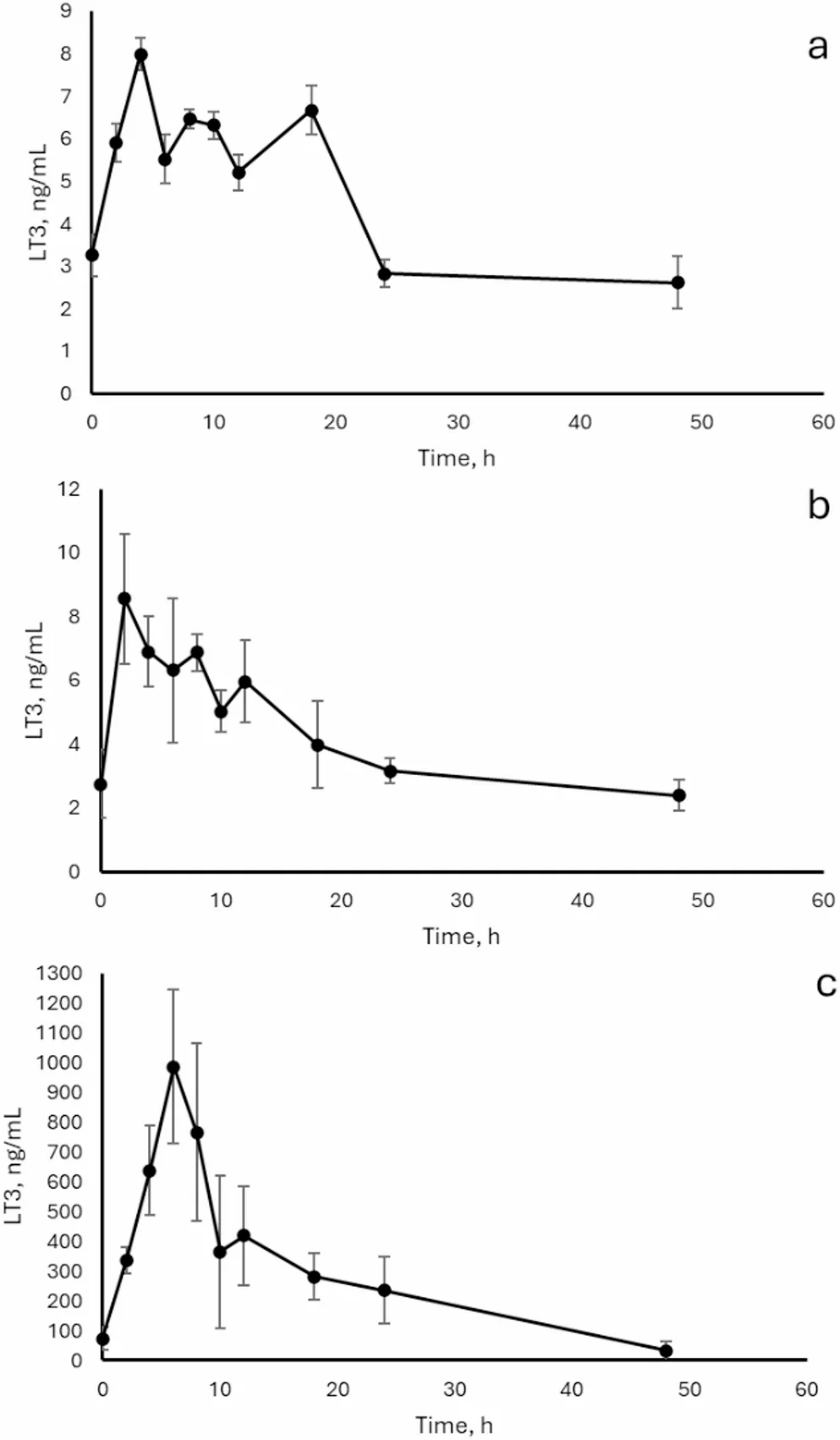
Pharmacokinetic profile of LT_3_ delivered by MNPs M, N and O in hypothyroid rats (see Table 1). (**a**) MNP M (10 µg LT_3_). (**b**) MNP N (100 µg LT_3_). (**c**) MNP O (1000 µg LT_3_). LT_3_ concentration was measured by ELISA for MNP M and MNP N (**a** and **b**) and by Luminex multiplexed immunoassay for MNP O (**c**). Data represent mean ± SD of *n* = 3 replicates

After administration of MNPs with the low dose of LT_3_ (MNP M), we found that LT_3_ concentration in the serum rapidly increased to a C_max_ value of 8.00 ± 2.15 ng/mL at a T_max_ of 7.14 h (Fig. 7a and Table 2). LT_3_ concentration remained significantly elevated compared to time zero (*p* < 0.05, Mann-Whitney U Test) and did not significantly change through 18 h (*p* > 0.05, one-way ANOVA), but then returned to baseline i.e., not significantly different from LT_3_ levels before MNP application 24 h after dosing (*p* > 0.05, Mann-Whitney U Test).

**Table 2:** Pharmacokinetic parameters from LT3 MNPs administered to rats^1^

PK parameter^2^	MNP M (10 µg LT_3_)	MNP *N* (100 µg LT_3_)	MNP O (1000 µg LT_3_)
T_max_ (h)	7.14	5.22	7.33
C_max_ (ng/mL)	8.00	8.56	987
T_1/2_ (h)	22.2	17.1	9.4
AUC (ng h/mL)	202	194	13,545

^1^ Data are from Fig. 7. ^2^ C_max_: Peak LT_3_ concentration. T_max_: Time to C_max_. T_1/2_: Time to half of C_max_. AUC: Area under the curve

When MNPs with the medium dose of LT_3_ (MNP N) were administered to rats, the PK profile again rapidly rose to a C_max_ value of 8.56 ± 2.04 ng/mL at a T_max_ of 5.22 h (Fig. 7b and Table 2). LT_3_ concentration again remained elevated compared to zero time for 12 h (*p* = 0.0159, Mann-Whitney U Test), but this time the LT_3_ concentration progressively decreased over that time period (*p* = 0.015, one-way ANOVA), returning to base line at 18 h (*p* > 0.05, Mann-Whitney U Test).

The MNPs with the high dose of LT_3_ (MNP O) showed different behavior. These MNPs led to a slower rise of LT_3_, reaching a peak concentration at a T_max_ of 7.33 h (*p* = 0.036, Mann-Whitney U Test), after which LT_3_ concentration remain elevated compared to zero time until 12 h = 0.036, Mann-Whitney U Test) and reached baseline at 18 h (*p* > 0.05, Mann-Whitney U Test) (Fig. 7c and Table 2). Notably, the LT_3_ concentration in the serum was much higher, reaching a C_max_ of 987 ± 258 ng/mL.

## Discussion

This study demonstrates proof-of-concept that LT_3_ (i) can be integrated into MNPs, (ii) can be delivered to the skin *via* a MNP, (iii) can reach the systemic circulation after MNP delivery to the skin, and (iv) can remain at elevated concentration in the serum for up to a day. This is a first key step to establish the viability of LT_3_ MNPs for clinical use in hypothyroidism to normalize and maintain serum T_3_ levels at or near, sustained, physiologic levels.

### LT_3_ MNP formulation and delivery to skin

In this study, we found that a MN formulation based on a casting solution containing 5% (w/v) PVP (10 kDa) in water was effective for making LT_3_ MNPs, although other formulations tested had similar performance. LT_3_ MNs were strong enough to be inserted into skin and deliver LT_3_ upon dissolution in both pig skin ex vivo and rat skin in vivo. While rat skin is considerably thinner than human skin [42], pig skin is generally considered a good model for human skin [43]. This suggests that the LT_3_ MNPs developed here may also be functional in human skin. The delivery efficiency of LT_3_ into the skin was moderate (i.e., 29–71%), although such delivery efficiencies are commonly reported for MNP-based delivery [24]. While this efficiency of LT_3_ delivery might be acceptable for future clinical use, it can likely be increased through additional MNP development and/or the use of an applicator for deeper skin insertion. While applicators have better control and optimize force and velocity for microneedle insertion compared to thumb insertion, they also increase the cost, complexity, and bulkiness to the patch system.

Although we did not detect LT_3_ accumulation in the Franz cell receiver compartment during ex vivo studies, we hypothesized that with blood flow through the skin, LT_3_ could be taken up and detected systemically, and thus in vivo studies were pursued.

### Nonlinear PK of LT_3_ administered by MNP

It is notable that the low- and medium-dose LT_3_ MNPs (10 and 100 µg LT_3_, respectively) had similar PK (i.e., similar C_max_) despite administration of 10-fold different LT_3_ doses, indicating nonlinearity in the PK response. In contrast, the highest-dose LT_3_ MNPs administered a LT_3_ dose that was 100-fold higher than the low-dose LT_3_ MNPs and achieved a C_max_ that was roughly 100-fold higher too, indicating a roughly proportional relationship between PK profile and dose.

One explanation could be that the MNPs did not actually contain the intended LT_3_ doses (e.g., the low-dose and medium-dose LT_3_ MNPs instead contained similar doses). However, we directly measured the LT_3_ doses in these MNPs and found them to be close to their intended doses (Fig. 3), indicating that this explanation is not likely to be correct. It is also possible that loading MNs with different LT3 doses produced different LT3 crystal polymorphs or resulted in different material microstructure within the MNs; future studies should assess this possibility.

Another explanation is that there is a mistake with the PK data from the medium-dose LT_3_ MNPs. If the C_max_ for these MNPs had been about 10-fold higher, then all three MNPs would have roughly shown dose proportionality in their PK profiles. However, we saw no other indication that the LT_3_ measurements for the medium-dose group might be flawed. We collected the serum samples and measured the LT_3_ levels using identical methods for the low-dose and medium-dose groups.

As noted below, we used a different analytical method for the high-dose group, which suggests a third possible explanation indicating that the high-dose group measurement was flawed. However, we do not suspect this is the case because the assay we used is an established commercial assay that was run by a highly experienced operator.

A final possible explanation is based on prior data showing that the D3 enzyme found in skin converts T_3_ to an inactive metabolite, T_2_, although the exact location (e.g. dermis, epidermis), extent of expression, and physiologic role of expression requires further investigation [22, 44–46]. Nonetheless, it may be that when the MNPs released the low- and medium-dose levels of LT_3_, most of the drug was deactivated by D3 and just a small amount was able to reach the bloodstream. However, when the larger-dose LT_3_ MNP (1000 µg LT_3_) was used, the resulting high concentration of LT_3_ was able to overwhelm the enzyme, thereby enabling much more LT_3_ to get into the systemic circulation. Additional studies will be needed to further test this hypothesis and provide a better understanding of the nonlinear PK response to LT_3_ administered to the skin by MNP.

### Analytical methods used for LT_3_ assay in serum

LT_3_ concentration in samples extracted from MNPs were measured by HPLC, but LT_3_ concentration in serum was much lower and therefore required a more sensitive assay. LT_3_ concentrations in the serum of animals that received low- and medium-dose LT_3_ MNPs (10 and 100 µg LT_3_, respectively) fell in the functional range of the ELISA assay, allowing its use in this study. In contrast, LT_3_ concentrations in the high dose (1000 µg LT_3_) MNPs were too high for the ELISA assay and therefore required dilution to fall into the functional range. We found, however, that dilution, even with the diluent recommended by the manufacturer, did not give accurate results, even with control samples of known LT_3_ concentration (Fig. S5 in Supplemental Information). We therefore concluded that the ELISA assay could only be used with samples having an LT_3_ concentration in the functional range of the assay without dilution.

This led us to use a bead-based Luminex immunoassay that was able to measure LT_3_ concentration in the range found in serum samples from animals that had received the high-dose LT_3_ MNPs (Fig. S6 in Supplementary Information). For this reason, the LT_3_ PK data for the low- and medium-dose LT_3_ MNPs are based on ELISA assay, while the data for the high-dose LT_3_ MNPs are based on the Luminex assay.

### Rat model of hypothyroidism

Because serum T_3_ levels in healthy, euthyroid, control rats are normal, we used a hypothyroid rat model to suppress endogenous T_3_ levels and thereby enable more-sensitive measurement of LT_3_ levels in the serum, as recommended by the American Thyroid Association guidelines [32]. Induction and maintenance of hypothyroidism was stressful for several rats, which resulted in the loss of some animals (Fig. S7 in Supplementary Information).

Euthyroid, control rats should have serum T_3_ concentrations of approximately 0.5 ng/mL, while hypothyroid rats should have much lower serum T_3_ concentrations [47, 48]. Despite our rats otherwise showing signs of severe hypothyroidism (e.g., lethargy, decreased appetite, weight loss, and piloerection) [47, 48], many of these rats had baseline T_3_ measurements that were unexpectedly elevated, with average pre-treatment concentrations of 3.3–73.4 ng/mL. Notably, anesthesia and heparin can impact T_3_ measurement [32], and the impact of depilatory cream is unknown. This is of perhaps less significance for the goals of this study, which were to generate PK data, because LT_3_ levels were well above baseline after LT_3_ MNP administration.

### Study limitations and future work

This study had a number of limitations that should be addressed in future work. Our optimization of LT_3_ MNPs was sufficient for the goals of this study, but additional work including, for example, other formulation excipients, could increase LT_3_ delivery efficiency into the skin and modulate LT_3_ release from MNs to achieve zero-order delivery, more-extended delivery duration, or other characteristics that could benefit future therapies. While we used a preservative (sodium azide) to inhibit microbial degradation during in vitro skin permeation studies, skin degradation during the week-long studies cannot be ruled out. We didn’t, however, see evidence of skin degradation, e.g., the skin was visually intact at the end of the experiments and there was no sharp increase in transdermal permeation. Despite variability, we were able to conclude that there were not major differences between the release profiles of the two formulations and that release occurred mostly for 3 days and then stopped increasing. The hypothyroid animal model and the LT_3_ immunoassay method both had limitations discussed above, suggesting that other animal models and other LT_3_ assays should be considered in future studies. While the PK profiles provided promising initial data, a better understanding of their nonlinear dependence on dose warrants further study. These studies in rats should be followed with additional optimization studies in larger cohorts of animals, including a concomitant oral LT3 control group and eventually in human subjects, evaluating both PK and pharmacodynamics to more fully assess the future use of a LT_3_ MNP for the treatment of hypothyroidism.

## Conclusions

This study presents a first assessment of MNPs for delivery of LT_3_, which could enable improved treatment of hypothyroidism. After screening a series of formulations for casting MNPs, we identified a PVP-based formulation that we used to administer various doses of LT_3_ to the skin, demonstrating effective skin penetration and delivery efficiency. PK studies in hypothyroid rats using three different LT_3_ doses spanning two orders of magnitude showed sustained LT_3_ concentrations in the serum for up to 24 h in a nonlinear, dose-dependent manner. These data support the further development of MNPs for LT_3_ delivery as an improvement over current thyroid hormone replacement therapy options for treatment of hypothyroidism.

## Supplementary information

Below is the link to the electronic supplementary material.

Supplementary Material 1

## Data Availability

All data and materials used in the study are presented in the main manuscript and in the Supplementary Information.
