# Supplementary material for: Delivery of liothyronine by microneedle patch for the treatment of hypothyroidism
Source: Drug Deliv Transl Res. 2026 Sep 2;16(11):4382–94. doi: 10.1007/s13346-026-02214-6 (PMC13748095; doi:10.1007/s13346-026-02214-6)
Supplement: Supplementary file 1 [file 13346_2026_2214_MOESM1_ESM.pdf]

SUPPLEMENTARY INFORMATION

Delivery of Liothyronine by Microneedle Patch for the Treatment of Hypothyroidism

Yasmine Gomaa[1,2], Tasnuba Hasin[1], Martin N. Griffin[3], Thy C. Nguyen[4], Richard K. Noel[5], Laura O'Farrell[5], Elizabeth A. McAninch[6,7], Ann Marie Kaiser[7], Levi B. Wood[3], and Mark R. Prausnitz[1,*]

[1] School of Chemical and Biomolecular Engineering, Georgia Institute of Technology, Atlanta, GA, USA.

[2]Department of Pharmaceutics, Faculty of Pharmacy, Alexandria University, El-Khartoum Square, Alexandria, Egypt

[3] George W. Woodruff School of Mechanical Engineering, Georgia Institute of Technology, Atlanta, GA, USA.

[4] School of Chemistry and Biochemistry, Georgia Institute of Technology, Atlanta, GA USA

[5] Department of Animal Resources, Georgia Institute of Technology, Atlanta, GA, USA.

[6] Division of Endocrinology, Gerontology, and Metabolism, Department of Medicine, Stanford University School of Medicine, Stanford, CA, USA.

[7] Equilibrate Therapeutics, LLC, Santa Barbara, CA, USA.

* Corresponding author: prausnitz@gatech.edu

Note 1. Implantation of Temporary or Permanent Jugular Catheters

Jugular catheters were made from 25 cm lengths of silicone rubber tubing (0.51 mm ID, 0.94 mm OD, Silastic, Dow Corning, Midland, MI) with one end cut at approximately a 45-degree bevel. The non-beveled end was fitted over a blunt 23G hypodermic needle. A small dollop of silicone rubber elastomer (Silicone Clone FX-302-1, Factor II, Lakeside, AZ) was placed 32 mm from the beveled end where it vulcanized. For chronically implanted catheters, a length of 4-0 braided silk suture was tied around the catheter prior to placing the silicone rubber dollop. Catheters were wiped with isopropyl alcohol and autoclaved.

Rats were anesthetized with isoflurane and placed on a warmed bed in dorsal recumbency. Using aseptic methods, an approximately 5 mm incision was made through the skin over the right external jugular vein parallel to the trachea. The vein was cleared of overlying tissue by blunt dissection so that its insertion into the pectoral muscle was visible. Two ligatures of absorbable suture (Ethicon Inc., Raritan, NJ) were placed around the vein. With the vein ligated rostrally, a venotomy was made 5 mm cranial to the junction of the vein and the pectoral muscle. The catheter was prefilled with saline and inserted 32 mm into the venotomy. This length placed the catheter tip outside the right atrium of the heart, which is well suited for repeated sampling.

For catheters that were removed at the end of a long day of anesthesia, the incision was temporarily closed around the catheter with tissue adhesive. For chronically implanted catheters the tubing was tied in place using the attached suture and the uninserted end was plugged with a sterile gold wire contact (#19003-00, Fine Science Tools, Foster City, CA) and passed through a subcutaneous tunnel to a small stab incision between the scapulae. Skin over the main incision was closed with wound clips. The tubing protruding between the scapulae was trimmed to 2 cm and kept plugged with a gold contact. Rats were given an injection of 0.08 mg/kg Buprenorphine HCl in Polymer (Wedgewood Connect, Albany, OH), then allowed to recover from anesthesia under a heat lamp and returned to their home cages.

Chronically implanted catheters were flushed once daily with 100 U/ml heparinzed saline using aseptic technique and a sterile infuser made from a blunt 23G needle shaft, 23G needle and a 5 cm length of silicone tubing. Samples were withdrawn as indicated using an infuser and the catheter was flushed and locked with heparinized saline and plugged with a sterile gold contact.

For rats catheterized only during one long day under anesthesia, samples were withdrawn as described above and the catheter was pulled out prior to recovery as described above using the absorbable suture to ligate the vein to prevent bleeding. Subsequent samples were collected either under restraint or under anesthesia by making a small incision near the tip of the tail and massaging the tail from base to tip.

Note 2. Sample Preparation for Luminex Immunoassay

Samples were thawed on ice, then centrifuged at 15,493 g for 10 min at 4°C. Prior to loading into a 96-well plate, samples were vortexed for 30 s, briefly centrifuged at room temperature (20 – 25 ºC), and mixed by pipetting to ensure homogeneity. Aliquots were taken from the sample center to avoid settled components.

A two-day assay was conducted using the MILLIPLEX Rat Thyroid Magnetic Bead Panel from Millipore (Sigma-Aldrich, St. Louis, MO) with minor protocol modifications. All pipetting steps, except washes, were performed using a single-channel pipette for consistency. To start, 200 μL of phosphate-buffered saline containing 0.1% Tween-20 (PBST) was added to each well of a 96-well plate. The plate was then sealed, shaken at 750 RPM for 10 min, and then decanted. After shaking, residual liquid was removed by inverting and tapping the plate. Each well then received 29.7 μL of MILLIPLEX Assay Buffer, followed by 6.25 μL of Millipore serum matrix solution. Then, 1.55 μL of sample was added to designated wells, excluding background wells, after which 6.25 μL of horseradish peroxidase (HRP) conjugate was added to all wells. The bead mixture was added to all wells and vortexed after filling each column to minimize settling and aggregation. The plate was then sealed, wrapped in foil, and incubated for 18 h on a shaker at 750 RPM at room temperature.

Following overnight incubation, plates were washed 3 times with 200 μL of PBST per well. For each wash, plates were placed on a magnetic holder for 2 min, decanted, then shaken at 750 RPM for 30 s at room temperature. After the final wash, plates were placed on a magnetic holder and then decanted, after which 12.5 μL of 1x MILLIPLEX Detection Antibody was added to each well. Plates were sealed, wrapped in foil, and then shaken at 750 RPM for 1 h at room temperature. Without decanting, 12.5 μL of 1x Streptavidin-Phycoerythrin (SAPE) was added to each well. Plates were re-sealed, covered in foil, and shaken at 750 RPM for 30 min at room temperature. Another wash sequence identical to the earlier steps was performed to remove unbound SAPE. Finally, 75 μL of MILLIPLEX Drive Fluid was added to each well, and the plate was shaken for 5 min at 750 RPM. All plates were read using a MAGPIX system (Luminex, Austin, TX).

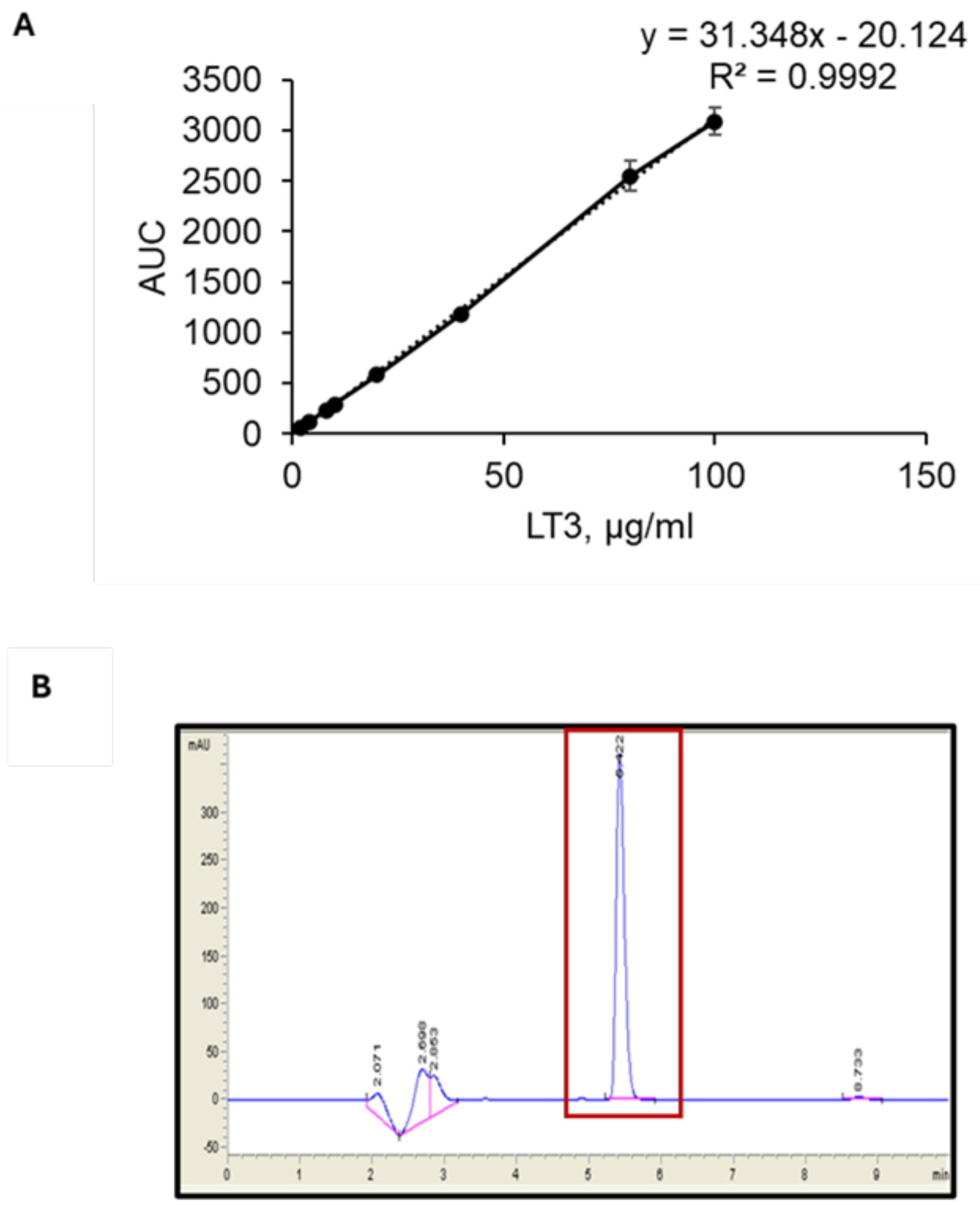

Fig. S1 HPLC analysis of LT_3. (A) Calibration curve of LT_3 over a range of 2 - 100 µg/mL. (B) HPLC chromatogram showing the elution peak of LT_3 (100 µg/mL) at a retention time of 5.4 min.

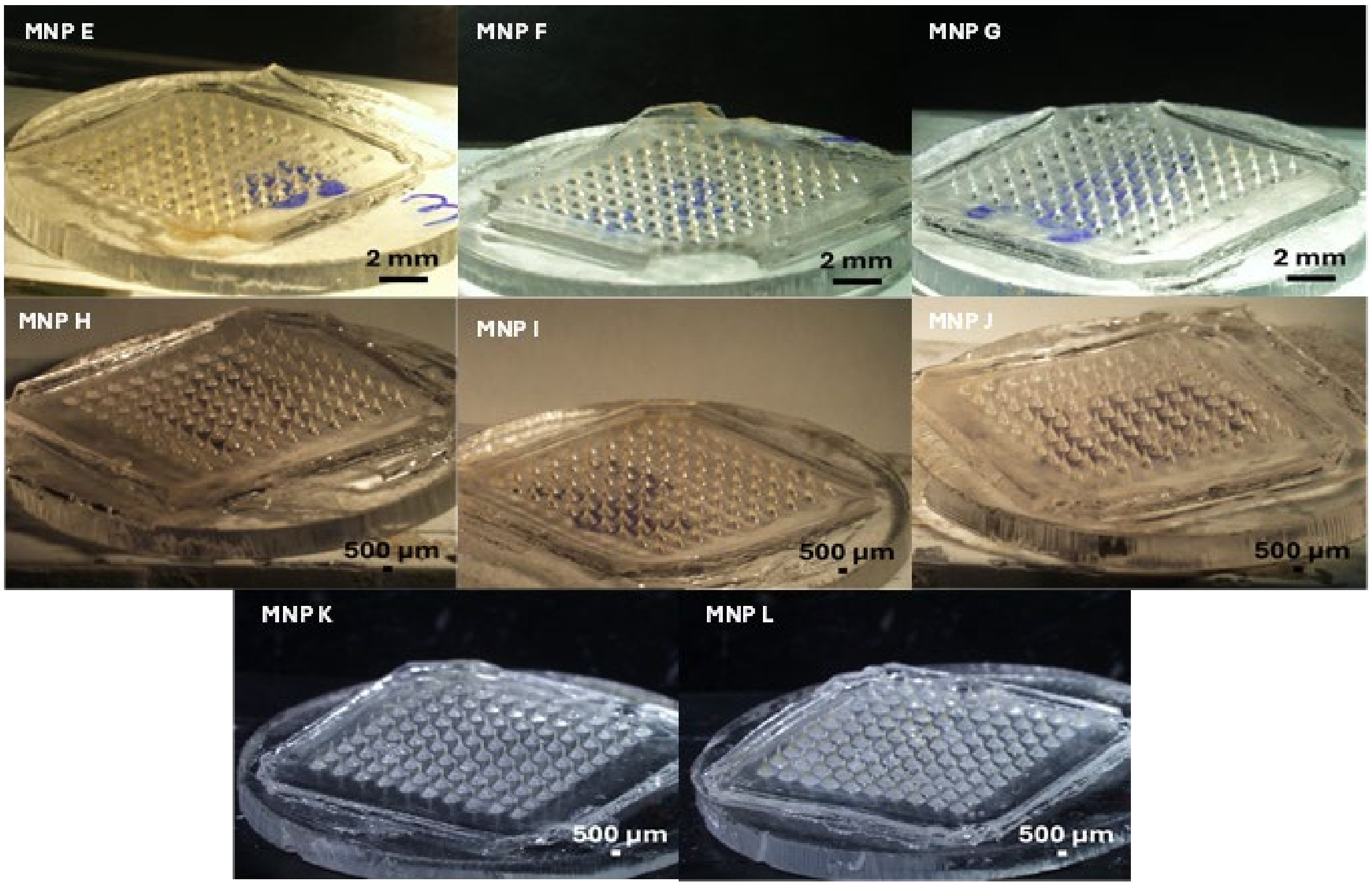

Fig. S2 MNPs containing LT_3. Representative MNPs are shown as imaged by light microscopy. MNP formulations were MNP E through MNP L (see **Table 1**).

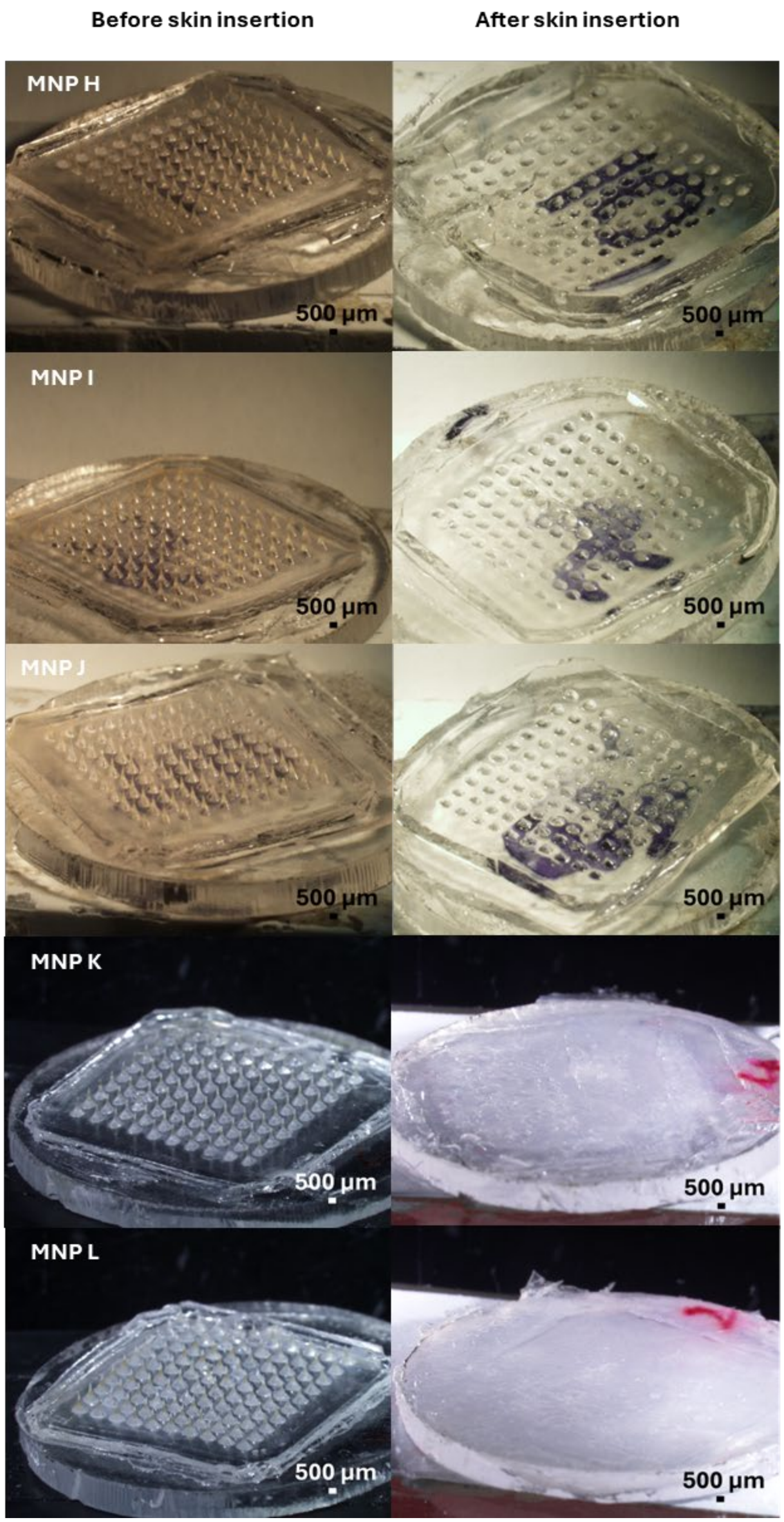

Fig. S3 LT_3 MNPs before and after insertion into pig skin ex vivo. Representative MNPs are shown before and after insertion into skin with 48 h (MNP H-J) and 1 week (MNP K and L) skin wear time. Imaging was performed by light microscopy. MNP formulations were MNP H-I (see **Table 1**).

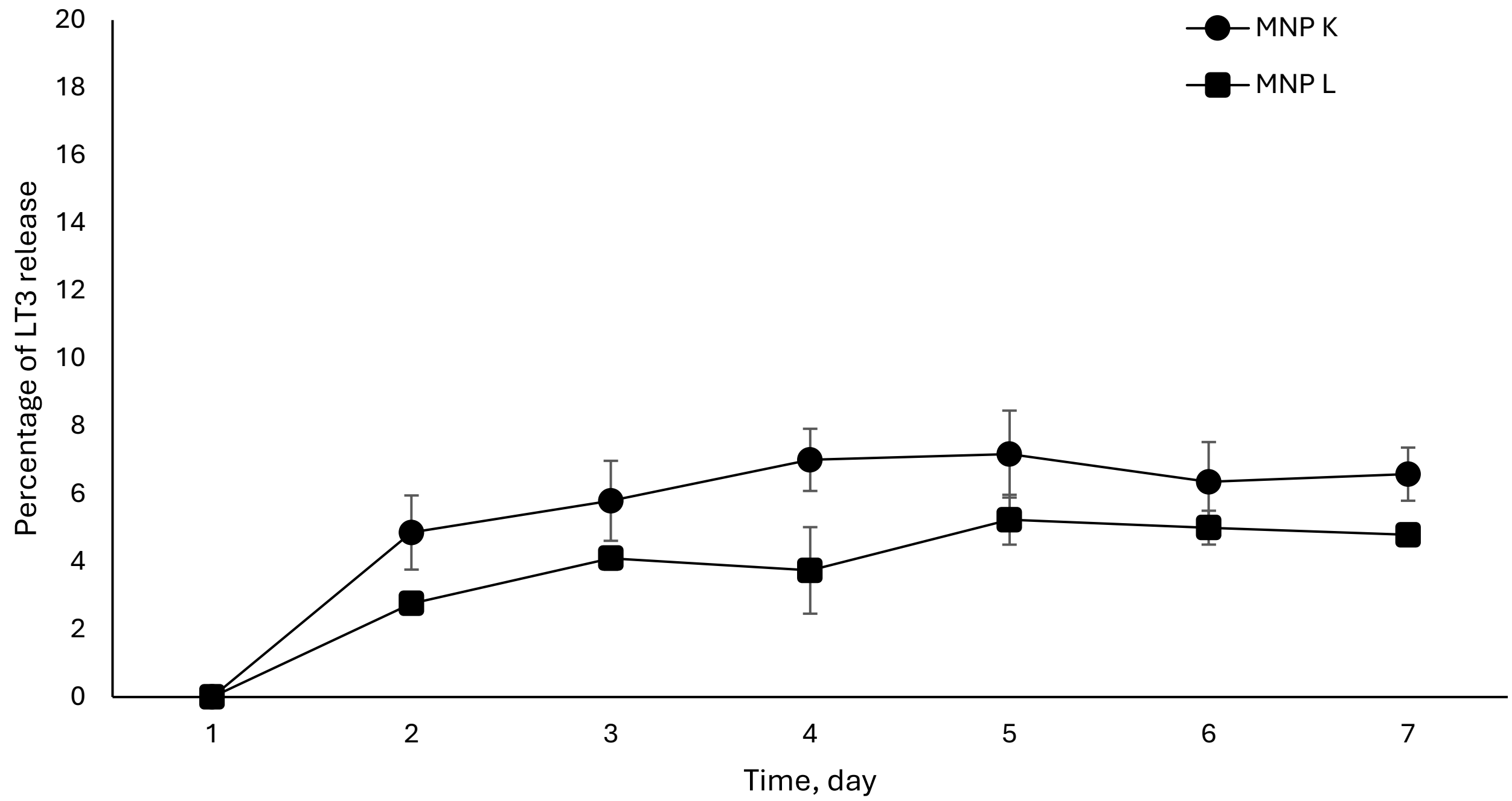

Fig. S4 Transdermal permeation of LT_3 through pig skin *ex vivo*. The cumulative amount of LT_3 permeated through skin after application of LT_3 MNPs. MNP formulations were MNP K and MNP L (see **Table 1**). Data represent mean ± SD of n = 3 replicates).

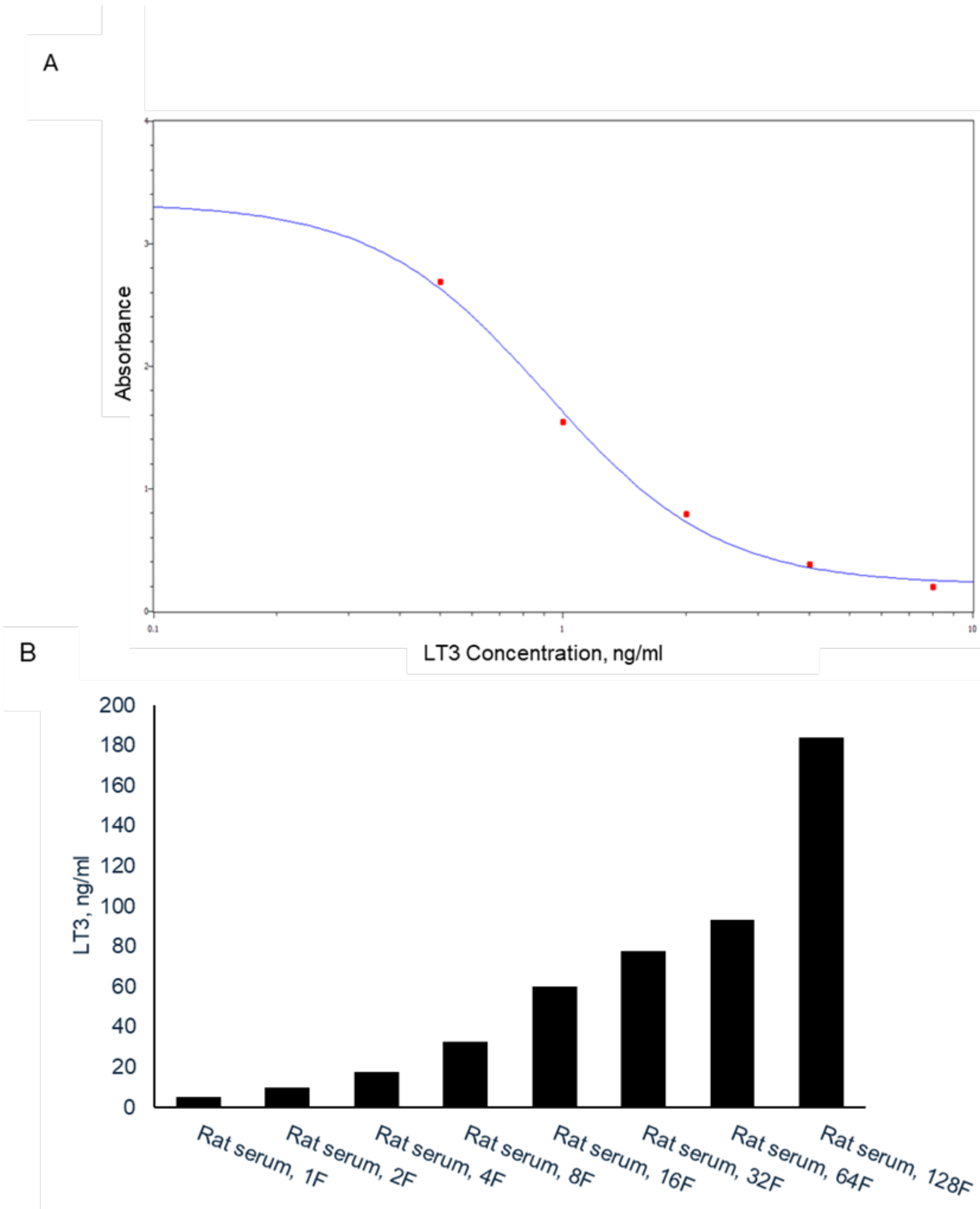

Fig. S5 Representative LT3 concentration measurements by ELISA. (A) Calibration curve of LT_3 concentration using the standards of known concentrations of the ELISA kit. (B) Effect of diluting rat serum on computed LT_3 concentrations using ELISA kit. The fold-dilution is indicated on the graph. LT_3 concentration calculations accounted for dilutions, meaning that the computed LT_3 concentrations for all samples should be the same.

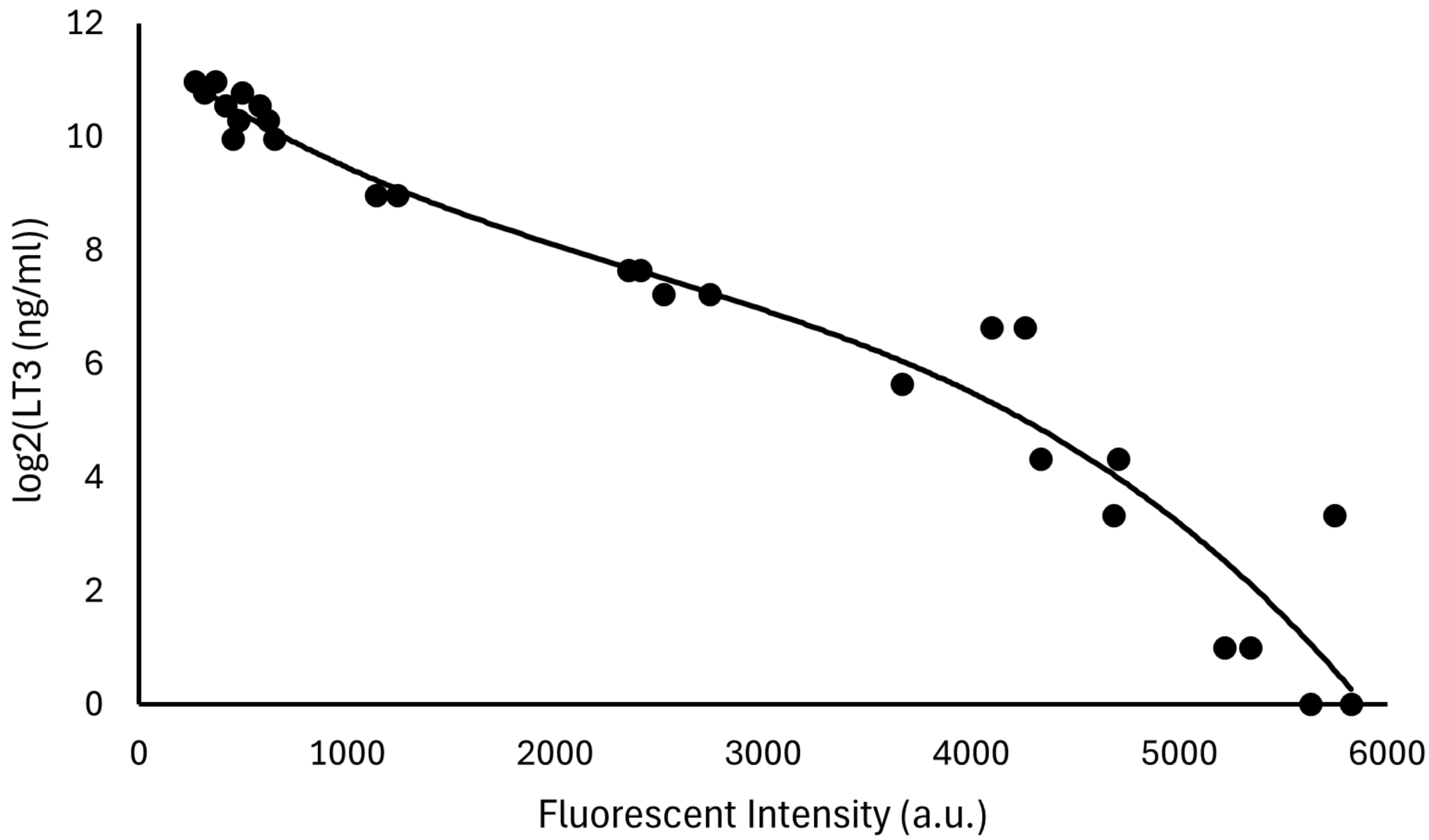

Fig. S6 Representative calibration curve of LT_3 concentration using Luminex multiplexed immunoassay. Calibration curve made using serum from naïve hypothyroid rats supplemented with increasing amounts of LT_3. Fluorescence intensity expressed in arbitrary units (a.u.). Calibration curve was fit to data using a local polynomial regression fitting in R.

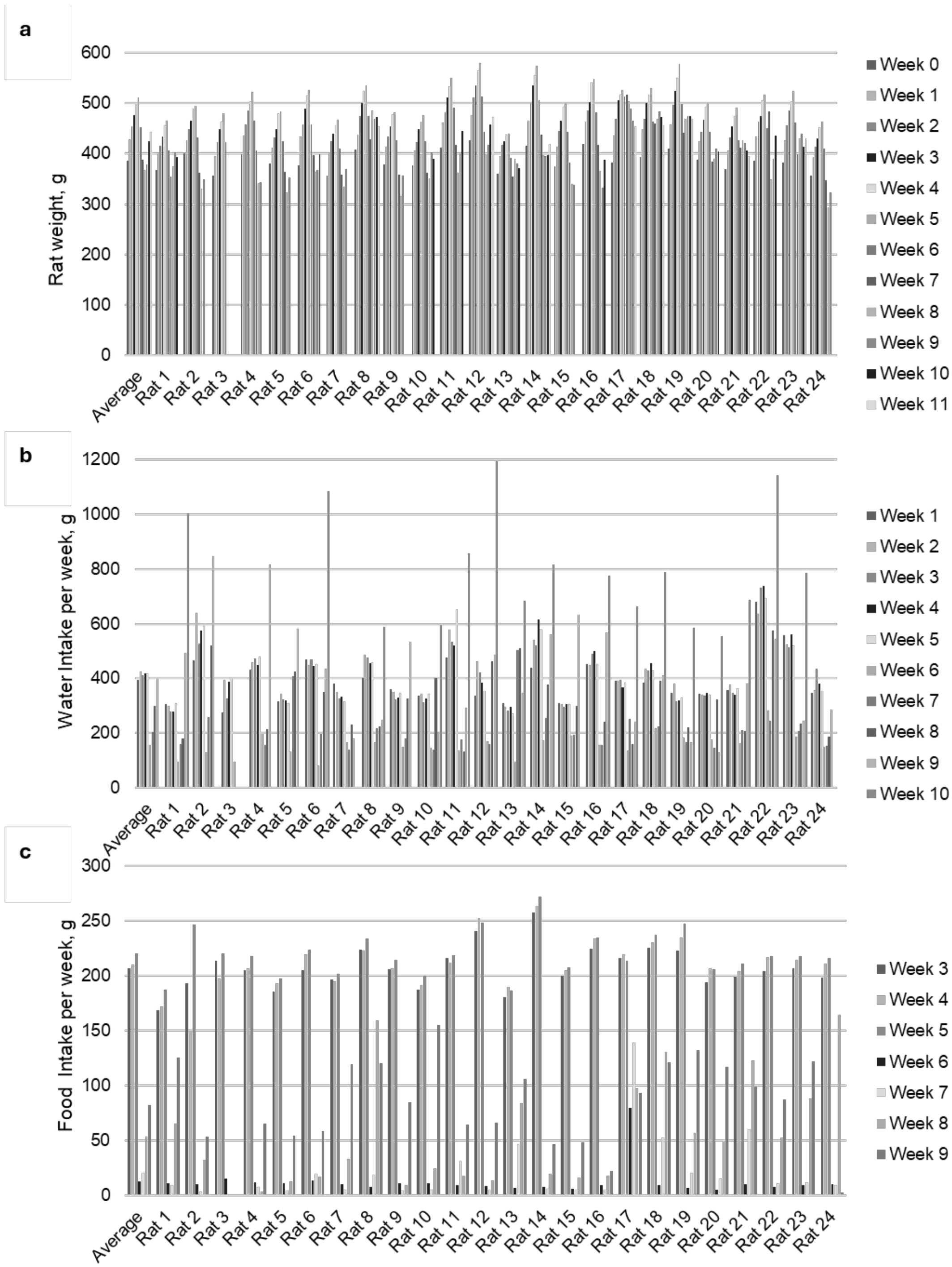

Fig. S7 Monitoring rats during pharmacokinetic study of LT_3 MNPs. (A) Rat weight. (B) Rat water intake. (C) Rat food intake. Rats 8, 12, 14, 17, 18,19, 21 and 23 received MNP M (10 µg LT_3), Rats 1, 6, 10, 11 (only t= 0 and 10 h), 13, 16, 20, and 22 received MNP N (100 µg LT_3), and Rats 2, 4, 5, 7, 9, and 24 received MNP O (1000 µg LT_3). MNPs were applied to rats on Week 9 for the 1000 µg LT_3 patch, week 10 for the 100 µg LT_3 patch, and week 11 for the 10 µg LT_3 patch. Rats 3 and 15 died during the hypothyroidism induction phase.
